# Proteome-Wide Mapping and Reverse Vaccinology Approaches to Design a Multi-Epitope Vaccine against *Clostridium perfringens*

**DOI:** 10.3390/vaccines9101079

**Published:** 2021-09-26

**Authors:** Fahad M. Aldakheel, Amna Abrar, Samman Munir, Sehar Aslam, Khaled S. Allemailem, Mohsin Khurshid, Usman Ali Ashfaq

**Affiliations:** 1 Department of Clinical Laboratory Sciences, College of Applied Medical Sciences, King Saud University, Riyadh 11564, Saudi Arabia; faldakheel@ksu.edu.sa; 2 Department of Bioinformatics and Biotechnology, Government College University, Faisalabad 38000, Pakistan; amnaabrar786@gmail.com (A.A.); sammanmunir01@gmail.com (S.M.); Seharaslam4376@yahoo.com (S.A.); 3 Department of Medical Laboratories, College of Applied Medical Sciences, Qassim University, Buraydah 51452, Saudi Arabia; k.allemailem@qu.edu.sa; 4 Department of Microbiology, Government College University, Faisalabad 38000, Pakistan; mohsin.mic@gmail.com

**Keywords:** subtractive proteomics, immuno-iformatics, sub-unit vaccine, *Clostridium perfringens*, molecular docking

## Abstract

*C. perfringens* is a highly versatile bacteria of livestock and humans, causing enteritis (a common food-borne illness in humans), enterotoxaemia (in which toxins are formed in the intestine which damage and destroy organs, i.e., the brain), and gangrene (wound infection). There is no particular cure for the toxins of *C. perfringens*. Supportive care (medical control of pain, intravenous fluids) is the standard treatment. Therefore, a multiple-epitope vaccine (MEV) should be designed to battle against *C. perfringens* infection. Furthermore, the main objective of this in silico investigation is to design an MEV that targets *C. perfringens*. For this purpose, we selected the top three proteins that were highly antigenic using immuno-informatics approaches, including molecular docking. B-cells, IFN-gamma, and T cells for target proteins were predicted and the most conserved epitopes were selected for further investigation. For the development of the final MEV, epitopes of LBL5, CTL17, and HTL13 were linked to GPGPG, AAY, and KK linkers. The vaccine N-end was joined to an adjuvant through an EAAK linker to improve immunogenicity. After the attachment of linkers and adjuvants, the final construct was 415 amino acids. B-cell and IFN-gamma epitopes demonstrate that the model structure is enhanced for humoral and cellular immune responses. To validate the immunogenicity and safety of the final construct, various physicochemical properties, and other properties such as antigenicity and non-allergens, were evaluated. Furthermore, molecular docking was carried out for verification of vaccine compatibility with the receptor, evaluated in silico. Also, in silico cloning was employed for the verification of the proper expression and credibility of the construct.

## 1. Introduction

*Clostridium perfringens* (*C. perfringens*) is an anaerobic, saprophyte bacteria with a ubiquitous distribution of environments including food, faces, and normal animal intestinal microbiota, including Homo sapiens. Type A of *C. perfringens* causes gas gangrene in humans and animals. Moreover, this widely dispersed, Gram-positive, rapidly growing, and opportunistic bacterium has become one of the most common bacteria that cause various enteric diseases in humans and animals, including enteric and histotoxic infections, non-food diarrhea, enterocolitis, and food poisoning [1,2]. *C. perfringens* is responsible for causing a lot of fatal infections in people, with the production of toxins as the primary means of pathogenesis in different hosts. Moreover, genomic studies of publically accessible strains of C. perfringens have reported a considerable level of genomic variation regarding mobile elements, episome content, and size of chromosomes [3]. Preventive approaches including proper storage and cooking of food are more effective in the prevention of this bacterial infection, but common antibiotic therapy is available for epidemics and severe cases, including cephalosporins and penicillin respectively; however, these conventional treatment methods might not guarantee effective treatment and may leads to an antibiotic-resistant pathogens.. Therefore, we designed an MEV against *C. perfrigens*.

Novel therapeutic techniques are urgently required to battle against *C. perfringens* infections. In the present situation, the most favorable choice to permanently control emerging infectious diseases is vaccination [4,5]. Immunization and vaccination are highly effective methods that facilitate the immune- system in targeting and recognizing bacteria [5,6]. A subtractive genomic approach was utilized to study the genome core for the identification of appropriate vaccine candidates. Subtractive genomics is a computer-based approach for recognizing potential drug and vaccine targets by discarding proteins that are not helpful for drug and vaccine development [7]. For a variety of reasons, computational approaches are more competent than traditional methods, including safety, low cost, hypersensitivity, stability, specificity, and accuracy. The combination of immuno-informatics and subtractive genomics is currently making vaccines cost-effective and more attractive. The availability of a large amount of proteomic data allows us to anticipate bacterium proteins that are suitable for vaccine production [8,9]. The utilization of vaccine informatics and computational immunology methods to construct a multiple-epitope (MEV) vaccine that is non-allergenic, non-toxic, and free from fragments of unwanted peptides is getting popular, and is currently utilized prior to vaccinology experiments. These methods are effectively applied to different infectious, bacterial, and viral pathogens [10,11,12,13]. The key objective of immuno-informatics is to highlight safe, antigenic, and immunodominant epitopes, which could induce safe and strong immune responses against bacteria and fulfill all the standards of a good vaccine candidate [6,14].

**Figure 1:**
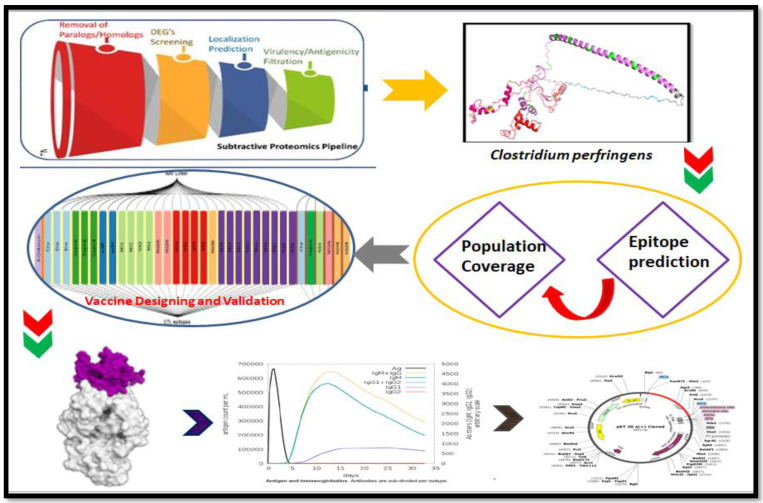
The Graphical abstract of the subtractive genomics-assisted pipeline utilized in this current study includes: whole proteome retrieval, removal of paralogs, essential gene retrieval, virulence factor identification, antigenicity prediction, subcellular location, CTL, HTL and B-cell epitope prediction and validation, vaccine construction, secondary and tertiary structure prediction and validation, molecular docking of MEV with TLR4 complex, MD simulation and immune-simulation, and in silico cloning.

The current study shows the screening of the entire proteome of *C. perfringens* by following groups of viral proteins. Each group of proteins was screened individually for the prediction of B- and T-cell epitopes together with their particular MHC alleles via vaccinomics. Thereafter, an MEV was constructed utilizing the more plausible epitopes with appropriate linkers and adjuvant. The linear sequence was utilized for immuno-informatics and physiochemical analysis, through 2D and 3D structure identification. The immuno-informatics vaccines have no or fewer side effects and are thermodynamically stable. Therefore, we next designed an effective MEV against *C. perfringens* and evaluated its efficacy and stability using various bioinformatics techniques, i.e., molecular docking of protein, PDBsum structure analysis, molecular dynamic (MD) simulation, immune simulation, and in silico cloning (computational expression validation). It was identified that the constructed MEV in our investigation is capable of creating strong interactions with receptors in the immune system, as well as initiating an effective host immunogenic response. The graphical representation of the methodology is shown below in Figure 1.

## 2. Methodology

### 2.1. Whole Proteome Retrieval

The whole *C. perfringens* (strain ATCC 13124/DSM 756/JCM 1290/NCIMB 6125/NCTC 8237/Type A) genome was downloaded from (proteome ID: UP000001823) UniProt and evaluated in a subtractive genomics approach to predict novel vaccine candidates [15]. Subtractive genomics is a computer-based technique for the identification of potential vaccine and drug targets through the removal of proteins that are not suitable for vaccine and drug construction [16]. The pathogenic genome has a paralogous sequence that arises due to duplication in evolution. CD–HIT was utilized to predict duplicated proteins in the genome having 90% identity to their sequence [17].

### 2.2. Essential Protein Retrieval

The proteome of the bacteria was subjected to a Geptop 0.5 server to identify the essential proteins of *C. perfringens* [18]. The essential proteins were evaluated further to discard proteins that were homologous to humans. BLASTp (NCBI) was used for screening of essential proteins at an e-value of 10^−4^ against Homo sapiens [19,20]. Homologous sequences among human and *C. perfringens* were removed to avoid an autoimmune reaction in the host.

### 2.3. Virulent Factor Identification

Virulent proteins perform an important pathogenic function and, therefore, are very vital in vaccine construction. Virulent proteins were predicted using the VFDB Virulent Factor Database). Homologs of VFDB labeled with a bit score of >100 and an identity of >30% for proteins of *C. perfringens* were regarded as virulent [21].

### 2.4. Antigenicity Prediction

Antigenicity is also defined as the ability to react quickly and be resistant to an antigen. Thus, it is crucial to choose a protein with greater levels of antigenicity in the development of peptide vaccines. All proteins of *C. perfringens* have ultimately been subjected to the VaxiJen 2.0 server with a threshold of 0.5 [6,22].

### 2.5. Subcellular Location

Subcellular locations of non-homologs and essential proteins of *C. perfringens* were predicted through the PSORTb and CELLO servers using a support-vector machine (SVM) based method [23,24].

### 2.6. CTL Epitopes Prediction & Validation

In antigen-specific identification, CTL performs an important function, making cytotoxic T-cell epitopes crucial to rational vaccine design. An MHC-I binding recognition server was used to predict cytotoxic T-lymphocytes (12-mer) epitopes, which are also termed as MHC-class I (http://tools.immuneepitope.org/mhci/, accessed on 1 May 2021). Sequences of proteins were submitted in FASTA format, the consensus approach was selected for identification, Homo sapiens were chosen as origin species, and then all the available alleles were selected to predict epitopes. Epitopes showing a percentile rank of 2 were considered for further analysis as a low score indicates greater binding capability [25].

The MHC-I tool of IEDB was utilized for the evaluation of cytotoxic T-Cell epitopes [26]. The VaxiJen 2.0 server was employed for the identification of antigenicity and to evaluate the capacity of epitopes to prompt an immune response (Table 1). The components of the vaccine must not present allergic responses [22]. Toxic and allergic responses must be prevented; simultaneously, immunogenic and antigenic candidates must be utilized for the construction of vaccines. For that, allergenicity recognition was performed using the AllerTop 2.0 web server, which uses the K-nearest neighbor approach for prediction [27]. The ToxinPred server was utilized for the identification of nontoxic CTL epitopes [28].

### 2.7. HTL Epitope Prediction & Validation

T helper (HTL) cells are a vital component of the adaptive immune system and can produce a cellular and humoral immune response against a foreign substance (antigen). Thus, MHC II alleles bound to Helper T-cells (HTLs) are crucial in vaccine design [29]. T lymphocytes boost B-cells against macrophagocyte bacteria to produce antibodies to destroy a parasite’s active cell [30]. The selected protein sequences were then submitted to the IEDB tool to predict HTL (15-mer) epitopes, utilizing the consensus method with corresponding alleles at a threshold value of 2 [31]. Helper T-cells secrete different cytokines including interleukin-10 (IL-10), interleukin-4 (IL-4), and interferon-gamma (IFN-gamma), which result in the activation of CTL and immune responses of other cells [32]. HTL epitopes including cytokines are important for the construction of vaccines. IFN-Gamma epitopes were predicted through the IFNepitope server using an SVM approach, IFN-Gamma vs non-IFN-Gamma model, and hybrid motif (Table 2).

### 2.8. B-Cell Epitope Prediction & Validation

In the initiation of an adaptive immunological response, B-cells perform an essential role and are therefore considered important building blocks of vaccines [33]. The ABCPred server was used to identify epitopes of B-cells [34]. A neural network-based approach is employed by ABCPred for the recognition of B-cell linear epitopes at a threshold of 0.5 (Table 3). Furthermore, the forecasted epitopes of B-cells were screened through ToxinPred, AllergenFP v.1.0, and VaxiJen for the evaluation of toxicity, allergenicity, and antigenicity, respectively [22,27,35].

### 2.9. Population Coverage

The distribution and expression of HLA alleles show variation due to region and ethnicity around the globe, therefore influencing the construction of effective epitope vaccines [36]. The population coverage server of IEDB was employed for computing the population coverage of chosen MHC-II and MHC-I epitopes, and for this purpose, all HLA alleles were examined [37]. On the basis of the distribution of Homo sapiens MHC binding alleles among different regions of the globe, population coverage was predicted.

### 2.10. Construction of Vaccines

An MEV was constructed by joining an adjuvant with epitopes of MHC-II, MHC-I, and B-cells with appropriate linkers. Adjuvants are immunogenic substances that can boost vaccine immunogenicity and therefore must be selected carefully [38]. Peptides selected for vaccine design are themselves not usually rich in immunogenicity. The adjuvant selected for MEV is choleragen chain B; the EAAAK linker allows the domains of a bifunctional fusion protein to be efficiently separated, allowing the first CTL epitope and adjuvant to be joined [39]. The linker used to integrate two epitopes is necessary for the epitopes to function effectively. To effectively detect epitopes in the vaccine, AAY and GPGPG linkers were employed for integration of CTL and HTL epitopes, respectively. The LBL epitopes were linked along with bi-lysine (KK) linkers to retain their separate immunogenic activity, as reported previously.

### 2.11. MEV Structure Analysis

Initially, Blastp screening against the human proteome was carried out to ensure a non-homologous MEV sequence [40]. The physiochemical properties including in vitro and in vivo half-life GRAVY (grand average of hydropathicity), AI (aliphatic index), II (instability index), theoretical IP (isoelectric point), and MW (molecular weight) of the constructed MEV was recognized through the ProtParam server [41]. Moreover, immunogenicity and antigenicity profiles were screened through the IEDB immunogenicity tool and VaxiJen 2.0 server, respectively [22]. The vaccine candidates must be non-allergenic; due to this, the allergenic elements of our constructed vaccine were computed through the AllerTOP tool [27]. The secondary structure of the MEV was predicted by SOMPA. This study also evaluates the various characteristics of the MEV, such as random coils, degree of beta turns, extended chains, and alpha helices [42].

### 2.12. 3D Structure Determination, Refinement, and Validation

3D structures, the lowest-energy protein structures, can twist and fold appropriately to form confirmations with greatest stability. For the construction of 3D structures, the I-TASSER tool was accessed for the designing of vaccines; it is used as a composite approach that exchanges information to enhance protective structure function identification and accuracy [43]. Refinement and optimization of the tertiary protein structure of the MEV was performed using the GalaxyRefine tool [44]. The overall relaxation of the structure is affected by MD simulation. A RAMPAGE tool study was performed using Ramachandran plots for validation of the MEV refined structure, by following ProSA-web server test of structure verification results for general score quality [45]. ERRAT was utilized to analyze dataset of non-bonded interactions in the vaccine construct of *C. perfrigens*.

### 2.13. B-Cell Epitope Screening

For the identification of conformational and linear B-cell epitopes of the designed vaccine, the Ellipro server of the IEDB and ABCPred tool was utilized, respectively. The amino-acid sequence of the vaccine was subjected to the ABCPred tool as input; an amino-acid length of 14 was selected, and the selected threshold was 0.5, while the tertiary structure of vaccine was submitted as input to the Ellipro server with the default parameters selected. The molecular-graphic v.13 system PyMOL was accessed to view epitope discontinuity in the resultant vaccine design [46].

### 2.14. Disulfide Engineering

The constructed vaccine model stability needed to be enhanced before further analysis. Disulfide bridges are covalent bonds that mimic stable molecular connections which, through the accuracy of geometric conformations, guarantee the protein model is significantly stable. Disulfide engineering is a novel method of forming disulfide links in a targeted protein structure. Therefore, engineering of disulfide bonds was performed through Design 2.0, and the refined structure of the vaccine was submitted as input. Initially, the refined structure of the vaccine was submitted and searched for residue pairings that might be employed in the engineering of disulfide bonds. Three residue pairs were chosen for their mutations in residues of cysteine through the mutated tool function [47].

### 2.15. MEV Docking with TLR4 Receptors

An efficient immune response is activated when an MEV protein interacts with the immune cells of the host. A molecular docking study was performed to check vaccine potential with Homo sapiens immune receptors. TLR-4 performs an essential part in human defense mechanisms, is a member of the pattern identification family of receptors, and reacts against high-response infections and greatly selective bacteria [48,49]. TLR-4 is vulnerable against PAMPs (molecular pathogen-associated patterns) including LPS’s (lipopolysaccharides) and lipo-oligosaccharides. Moreover, TLR-4 determines molecular pathogen-associated patterns from fungi, viruses, and mycoplasmas. TLR-4 was carefully studied and researchers found it has an essential role in boosting anti-pathogenic reactions [50]. Thus, TLR-4 was selected as a receptor, and PDB (ID: 4G8A) was used to download its structure. For docking with multiple epitope vaccines, HADDOCK v2.2 tool was utilized [51]. High Ambiguity Driven protein-protein docking (HADDOCK), a flexible docking approach, was utilized for the construction of biomacromolecular complexes. The docking complex was visualized through the molecular graphic system PyMOL [52]. Furthermore, evaluation of interacting residues in docking complexes was done using the PDBsum online database [53].

### 2.16. MD Simulation

In any in silico investigation, the analysis of molecular dynamics is critical for determining protein–protein complex stability. The stability of proteins can be determined through the comparison of key protein dynamics with their normal modes [54]. The iMODS tool was used to perform normal mode analysis on the collective protein motion within the internal coordinates [55]. The tool estimated B-factors, covariance, levels of intrinsic motion of the complex with regards to eigenvalues, deformability, and paths. The primary chain’s deformability is determined by whether each of its residues is capable of deforming a certain molecule. Stiffness of motion is described by the value of the normal mode; this is closely related to the energy required to deform structures, therefore deforming structures with a low eigenvalue is considerably easier.

### 2.17. Immune-Simulation

An in silico immune simulation was computed using the C-ImmSim 10.1 server to authenticate the planned MEV immunological responses. C-ImmSim simulates the three main components of the functioning mammalian system (thymus, lymph node, and bone marrow) [56]. The parameters chosen for the input of immune simulation were selected as default.

### 2.18. In Silico Cloning & Codon Optimization

Codon usage within organisms differs according to species, therefore an unadapted codon can cause a low expression rate within the host. The sequence of amino acids was reverse-translated and improved by utilizing the JCAT (java codon adaption tool) tool to adjust our codon vaccination to *Escherichia coli* (strain K12), a popular prokaryotic model [57]. Rho-independent transcription termination ribosome binding-sites of prokaryotes were chosen as enzyme restriction cleavage sites. The online web server calculated the CAI (codon adaption index) and GC content for an improved sequence of nucleotides that represent expression levels in *Escherichia coli* (strain K12) [58]. Finally, the two compatible restriction sites of enzyme Xhol and Ncol were introduced at both ends of multiple-epitope sequence for its in silico cloning within a pET 30a plasmid (+), by utilizing the SnapGene software (https://www.snapgene.com/, accessed on 28 July 2021); several studies have used this vector for cloning [59,60].

## 3. Results

### 3.1. Protein Selection

The entire genome of *C. perfringens* (strain ATCC 13124/DSM 756/JCM 1290/NCIMB 6125/NCTC 8237/Type A) has a total of 2875 proteins. Out of these, 330 were considered essential proteins using the Geptop 0.5 online tool. Homo sapiens paralogos were discarded after BLASTp analysis and 109 proteins were identified as non- homologous; these were evaluated on the basis of antigenicity values. The best three proteins with top antigenicity values that exhibited extracellular localization were selected.

### 3.2. Evaluation and Identification of B- and T-Cell Epitopes

From the *C. perfringens* target protein, 17 epitopes of CTL (12-mer) were identified. Their antigenicity, toxicity, and allergenicity were assessed and selected for the vaccine design (Table 1). In the same way, three epitopes of HTL were predicted (Table 2). The cytokine-stimulating abilities of HTL epitopes were computed and chosen for construction of the vaccine. Similarly, five B-cell epitopes were chosen for the construction of the vaccine, having greater antigenicity and being non-allergenic and nontoxic (Table 3).

### 3.3. Population Coverage

**Figure 2:**
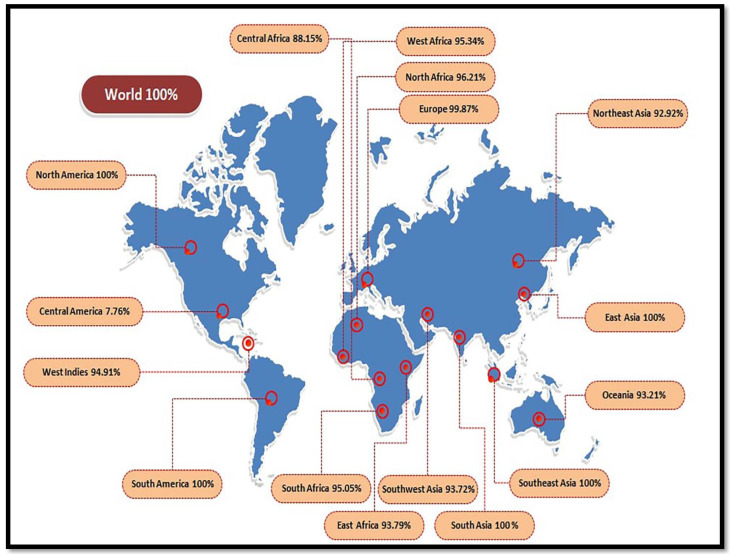
Population coverage analysis of alleles around the globe.

The frequency of the HLA allele changes among various geographical populations and ethnicities across the globe, which makes population coverage a vital parameter in the designing of the vaccine. In this study, the shared population coverage of selected epitopes with their associated allele of HLA was computed. This study represented the collective coverage of the global population for chosen epitopes of ~100% coverage. The maximum population coverage was recorded in South Asia, East Asia, and South East Asia, with a collective coverage of 100%. The lowest population coverage was reported in Central America (Figure 2). In brief, our investigation confirmed that the epitopes chosen would be good candidates for the construction of an MEV.

### 3.4. Multiple Epitope Vaccine Construction

**Figure 3:**
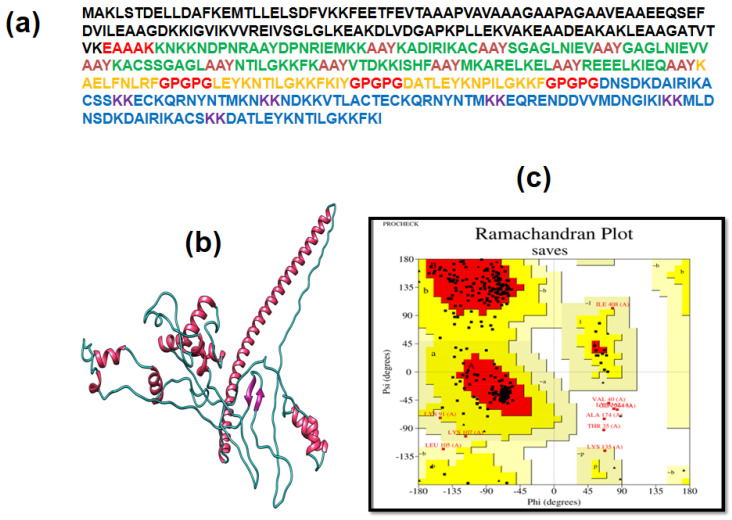
(**a**) Multiple epitope vaccine sequence structure (adjuvant, EAAK, CTL, AAY, GPGPG, HTL, LBL, and KK were labeled with black, red, green, hot pink, red, orange, blue, and dark purple respectively). (**b**) Refined structure of MEV 3D structure (helix, beta strands, and coils are represented in hot pink, purple, and cyan). (**c**) Ramachandran plot analysis of Saves shows that 86.6% residues are within a favorable region.

A multiple epitope was constructed using all epitopes that were selected. All epitopes of LBL, HTL, and CTL were combined by KK, GPGPG, and AAY linkers, respectively (Figure 3a). These linkers are beneficial because they enhance immunization and epitope performance while simultaneously inhibiting the development of junctional epitopes [61]. Moreover, B-chains of the Cholera enterotoxin (124 bp), acting as an adjuvant for the final vaccine design, was further joined at the N-terminal through an EAAAK linker (Figure 3a). The EAAAK linker was utilized because it enhances overall structural stability and deceases associations with other protein regions through effective detachment [62]. The final constructed multiple epitope vaccine presented 415 amino acids (Figure 3), exhibiting different epitope arrangements with their linkers.

### 3.5. Immunogenic and Physicochemical Analysis

After the development of the vaccine structure, its physiochemical and immunogenic properties were studied. Initially, homologous analysis of the designed vaccine was estimated against the Homo sapiens proteome, and outcomes validated that it has no homology with any area of the Homo sapiens genome. Furthermore, the toxicity, allergenicity, and antigenicity of the MEV model were calculated. Results exhibited that our MEV model is highly non-toxic, non-allergenic, and antigenic (0.82 at 0.50 threshold). Afterward, the physiochemical characteristics of the designed vaccine were predicted through ProtParam. The MW and theoretical PI of the constructed vaccine were 4507.10 Da and 9.23, respectively. The GRAVY (grand average hydropathicity) was −0.453; the negative sign represents the hydrophilic nature of MEV. The half-life means of our designed MEV have been calculated to be, >20 h in yeast, (in vivo), and >10 h in *E. coli* in vivo and 30 h in vitro. All these results suggested that *C. perfringens* MEV can be considered as a possible vaccine candidate.

### 3.6. Analysis of Structure

SOPMA was utilized to investigate the vaccine secondary structure. According to this research, 38 amino acids (16.87%) in the entire vaccine formed extended beta strands, 109 amino acids (26.27%) formed the coils, and 198 amino acids (47.71%) created α-helix.

### 3.7. 3D Structure Determination, Refinement, and Verification

For tertiary structure identification of the MEV of *C. perfringens*, the I-TASSER tool was utilized (Figure 3b). The checkerboard score was −3.14 in the I-TASSER. The GalaxyRefine online tool was used to refine the forecasted structure. The Ramachandran plot estimated that 86.6% residues were within favorable region, 10.2% in the allowed region, and 1.6% in the disallowed region (Figure 3c). The resultant Z-score was 0.465. In ERRAT, the evaluation of the refined structure score was 80%. These results showed that the optimized structure was of good quality.

### 3.8. Screening of B-Cell Epitopes

Besides producing cytokines, B-cells also secrete antibodies, therefore providing humoral immunity [63]. Therefore, the designed vaccine structure must have ideal epitopes of B-cells. Six continuous/linear and eight discontinuous/conformational epitopes were identified from the structure of vaccine without changing the prediction factors in Ellipro and ABCPred 2.0. The visualization of the conformations of epitopes of B-cells in the tertiary structure of the constructed vaccine was done through the PyMOL molecular visualization system.

### 3.9. Disulfide Engineering

**Figure 4:**
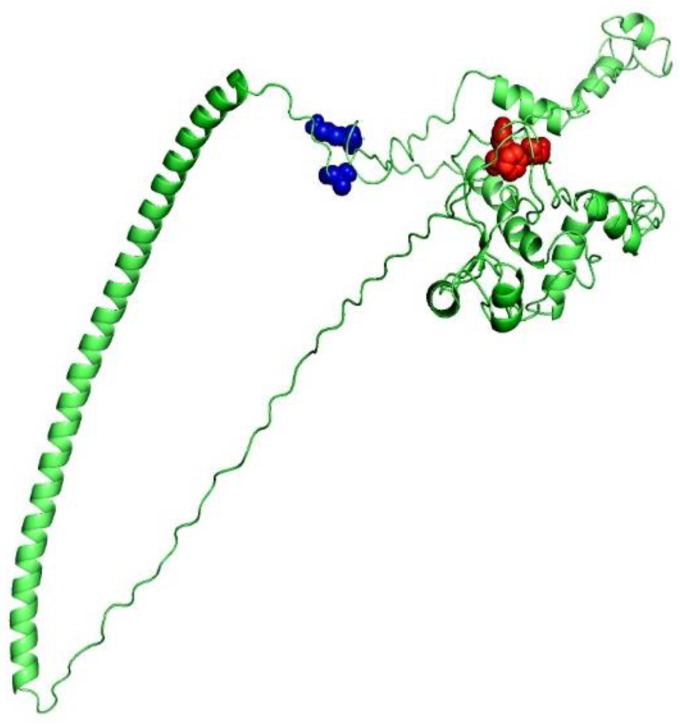
To improve the stability of the MEV, disulphide engineering was performed. The green color represents vaccine structure whereas the blue and red colors represent the two mutated pairs, selected on the basis on the X^3^ and B-factors.

DbD2 (Disulfide by Design 2.0), a web tool, was utilized for performing disulfide engineering to increase the refined vaccine construct stability. For the engineering of a disulfide bridge, 25-residue pairs can be employed. Two pairs of residues represented Chi3 value and energy in the usual range, and were hence selected for the purpose of disulphide engineering (Figure 4). Subsequently, four mutations were thus produced in the residue pair, i.e., C59E–C63F with an energy of +83.41 kcal/mol, and ALA167–GLY410 with an energy of −84.30 kcal/mol.

### 3.10. Docking between MEV & TLR4

**Figure 5:**
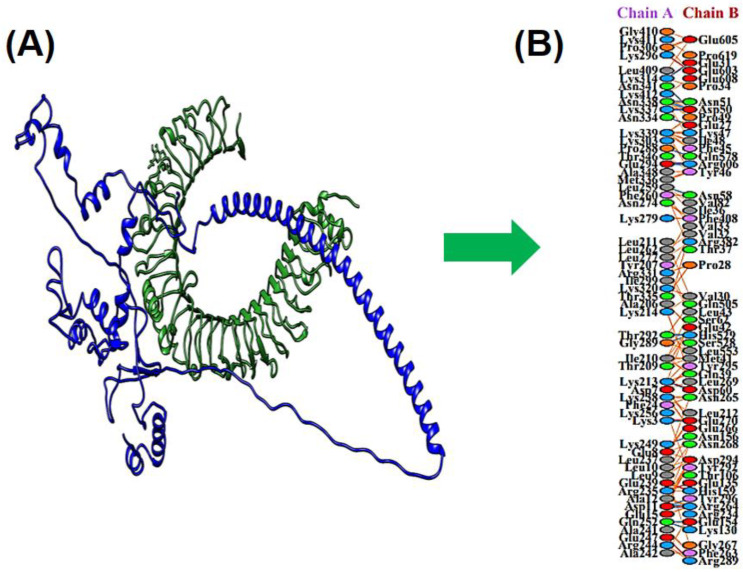
The TLR-4 is represented in green (Chain A) and the MEV is represented in blue color (Chain B). The section (**A**) in figure represent the vaccine and TLR-4 docking complex and the section (**B**) in figure represent interactions.

A suitable association between immune receptors and antigens is essential for triggering an immune reaction. Thus, the HADDOCK—v2.2 online tool was accessed for docking of constructed multiple epitope vaccines with Homo sapiens immune receptors. TLR-4 is capable of producing immune reactions effectively after pathogen recognition. The docking results showed that TLR-4 and MEV interact strongly (Figure 5). The binding score of MEV-TLR-4 was 84.2 ± 23.3 (Table 4).

### 3.11. Molecular Dynamics (MD) Simulation

**Figure 6:**
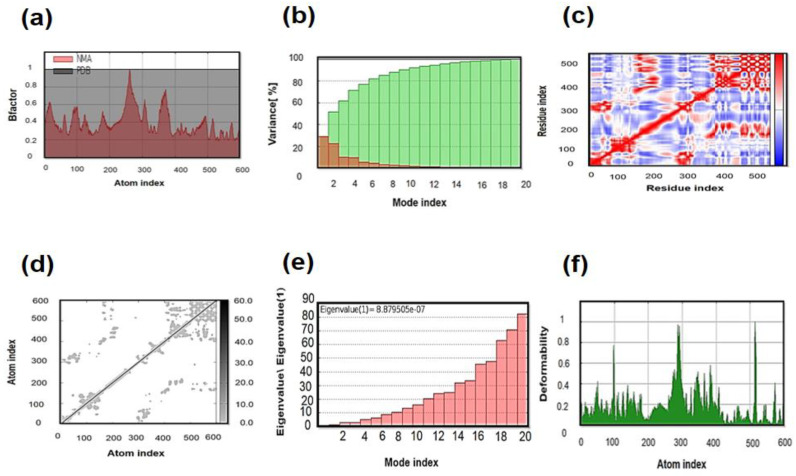
MD simulation of the MEV refined structure: (**a**) B-factor, (**b**) variance, (**c**) covariance matrix, (**d**) elastic network analysis, (**e**) eigenvalue and (**f**) deformability.

NMA (normal mode analysis) was performed to investigate the mobility of proteins along with their stabilization on a larger scale. iMODS, an online web tool, was employed for this assessment. The complex deformability of each residue is based on a single distortion, as represented by chain hinges. The suitable value obtained was 8.532549 × 10^−5^. The eigenvalue was the reverse of the variance that was related to each normal model [64]. The B-factor value as a result of normal mode analysis was RMS-proportional. The residue pair combinations are represented in white, red, and blue with individual pairs of anti-correlated, unconnected, and associated movements displayed in Figure 6. The elastic map depicts atom pairs connected by springs, with each point representing one spring, and a grey hue showing stiffer areas.

### 3.12. Immune Simulation

**Figure 7:**
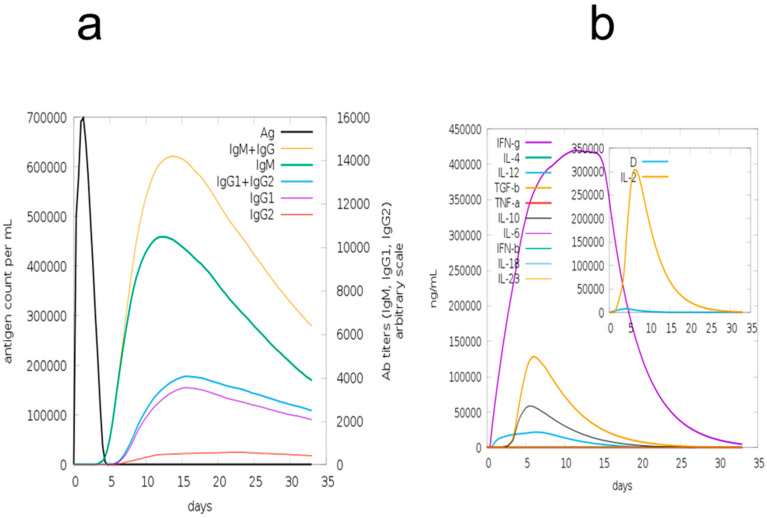
In silico immune reactions of multiple epitope-based vaccines from an antigen: (**a**) development of interleukins and cytokines; (**b**) immunoglobulin generation and isotopes of B-cells following exposure to the antigen in different states on the Simpson Index.

The primary and secondary immune responses play a significant role in bacteria, and probably in real immune responses. In silico responses of the host immunological system against antigens is represented in (Figure 7). In primary and secondary stages with instantaneous antigen decreases, the primary reaction was predicted to involve increased IgM and IgG + IgG levels, followed by IgG1, IgM, and IgG2 + IgG1. Moreover, robust cytokine, as well as interleukin, responses were detected. This shows the effective immune response of MEBV’s, along with their discharge upon successive encounters.

### 3.13. In Silico Cloning

**Figure 8:**
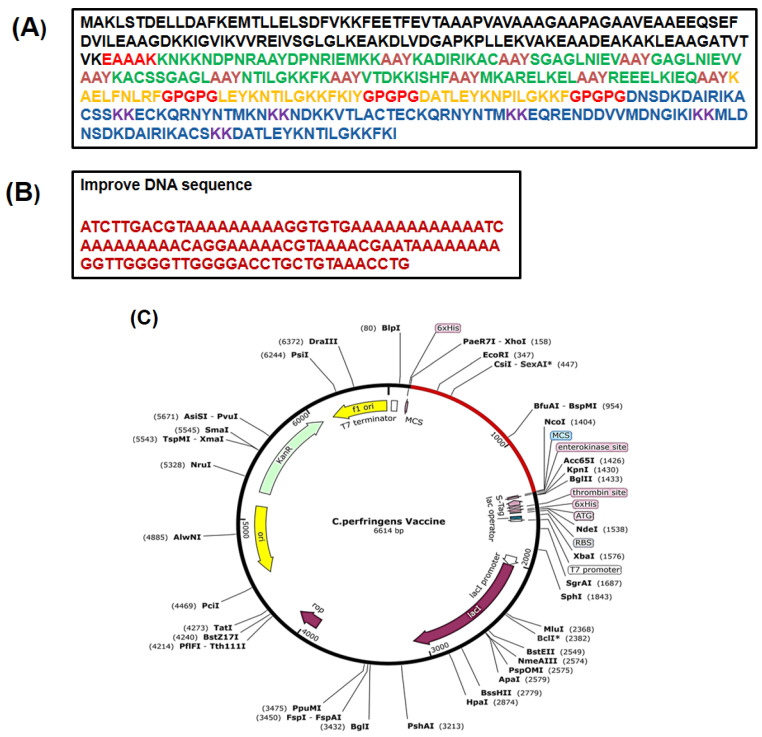
(**a**) Original sequence of MEV; (**b**) optimized DNA sequence; (**c**) codon-optimized MEBV was cloned into the *E. coli* K12 expression system in-silico. The backbone of the plasmid is shown in black, while the inserted DNA sequence is represented in red; * in-silico.

The key purpose of in silico cloning and codon optimization was the efficient expression of the protein within the *E. coli* host. Here, codons of *C. perfringens* present in the structure of the vaccine were modified in accordance with the K12 strain of *E. coli*. The DNA CAI score after adaptation was 1.0 and the GC-content of the DNA sequence was 45%. A score of CAI close to 1.0 suggested a satisfactory modification. The improved codon was introduced into pET 30a vector (+) of *E. coli* between Ncol and Xhol restriction sites, as represented in (Figure 8). Therefore, the total clone length was 6614 bp.

## 4. Discussion

*C. Perfringens*, a widely dispersed, Gram-positive, rapidly growing and opportunistic bacterium has become one of the most common bacteria that causes various enteric diseases in humans and animals, including enteric and histotoxic infections, non-food diarrhea, enterocolitis, and food poisoning [1,65]. Moreover, it causes gas gangrene in animals and humans [66]. The MEV discards non-essential elements, in contrast to traditional vaccines, which may cause abnormal reactions in the immune system or may have harmful effects [67]. Our main aim was to design a multiple epitope vaccine that may initiate a powerful immune reaction following vaccination, keeping all the benefits of multiple epitope vaccines in mind.

Investigators have tried to improve cost efficiency and reduce detrimental consequences and time for developing vaccines for a long time. There are presently various voluntary strategies that exist for the development of competent and effective novel generation MEVs that adopt techniques of immuno-informatics [68,69]. Immuno-informatics approaches are helping investigators by decreasing the burden of experiments in laboratories; furthermore, these techniques are low-cost, and less laborious in comparison to conventional techniques [70,71,72]. During the past decade, in silico drug design has made significant progress. Several biological problems have been solved by implementation of various bioinformatics techniques.

In the current study, the whole proteome of *C. perfrigens* was computed to the subtractive genomics pipeline to select the most suitable proteins for choosing appropriate epitopes and for MEV development. To our knowledge, this is the first study to design a possible vaccine candidate against *C. perfrigens*; Unni et al. anticipated possible epitopes that might be used for the construction of a successful *C. perfrigens* vaccination but did not suggest any vaccine structure [73]. Virulent antigenic proteins are possible targets for the construction of a computer-based vaccine. Bacteria infect their host by virulent proteins [74]. To avoid autoimmune responses, human homologs were identified and discarded. Furthermore, paralogous, cytoplasmic, and nonessential proteins were discarded because these are less significant. Because of their role in virulence and pathogenic adhesion to host cells, membrane and extracellular proteins were considered for choosing epitopes and for effective MEV construction [6]. The top three essential proteins that were important for antigen and pathogen survival were selected as potential candidates for the vaccine. The chosen proteins were then further utilized for the prediction of epitopes.

To predict the epitopes of HTL, LBL, and CTL and to choose appropriate vaccine candidates, different databases and online servers were utilized [75]. Helper T lymphocyte initiate both humoral and cell-mediated immune reactions, cytotoxic T lymphocytes prevent the virus from spreading by killing virally infected cells and by producing antiviral cytokines, and LBLs are responsible for the development of antibodies [76]. Because of their greater significance is inducing in immune responses, the final MEV structure was identified for both T- and B-cell cell epitopes. The key factors that were considered for the selection of the best epitopes were immunogenicity, toxicity, allergenicity, and antigenicity. Helper T-cells have the ability to produce cytokines, e.g., interferon-gamma, and have a greater tendency to reduce proinflammatory reactions, which results in decreased tissue damage. HTL can also help in the stimulation of CTLs. To combine epitopes of LBL, CTL, and HTL, KK, AAY, and GPGPG linkers were utilized. Linkers are utilized to enhance the folding, stabilization, and expression of the MEV [77]. Adjuvants require much attention due to their control over humoral and cellular responses in immune reactions [78]. In the construction or designing of a vaccine, adjuvants boost durability, influence stability, immune response, and antigen growth, and protect against pathogen infection [79].

When assessing the MEV construct, it was found, by using the Vaxijen tool, that the vaccine structure represented more antigenicity than the nonadjuvant model, clearly representing that that particular adjuvant has a significant role in chimeras. The MW of the vaccine construct is approximately 4507.10 Da, showing the reliability of MW with regards to the MEV. The MEV is considered to be easily available and soluble within the host [80]. The numerical value of PI reveals the vaccine’s basic nature. Furthermore, the predicted instability index shows the stability of the proteins even after they have been expressed, and thus enhances its potential for use in further studies.

Thermostability and hydrostability are correspondingly represented by the aliphatic index and GRAVY score. The MEV half-life mean is >0 h in yeast, 30 h in vitro, and >20 h in vivo, which is in line with previously reported data [81,82,83]. Furthermore, the MEV has been represented as non-allergenic, highly antigenic, immunogenic, non-toxic, and flexible. These results show that vaccine constructs have the ability to create a true immune response and prevent side-effects. 3D structure identification provides detailed knowledge of spatial models of key elements of proteins and helps in the studyof other elements of proteins: their interactions with ligands, dynamics, and functions [84]. The required properties of a multiple-epitope based vaccine were studied by 3D structure modeling. Several different computer-based approaches were employed, and the outcome showed the overall high quality of the identified structure.

The results showed that the selected epitopes and their respective alleles perfectly cover a number of geographical areas across the whole world. The greatest population coverage was observed in South Asia, East Asia, and South East Asia, with a collective coverage of 100%; these are the regions where the most significant epidemics of *C. perfringens* have. Hence, in these geographic areas, candidate for vaccines are vital to protect individuals against *C. perfringens* infections. The derived data was subjected to molecular dynamic simulation and protein-ligand docking analysis to calculate a potential immune response and to check the stability between the protein and TLR-4, considering the utilization of TLR-4 as a potential adjuvant for the designed chimera.

The minimization of energy was performed to lessen the binding energy (BE) of the entire system for the overall stabilized conformation of the TLR4-protein docked complex. The minimization of BE lessens the improper geometric structure through the replacement of respective protein molecules, therefore increasing the stability of the structure with suitable stereochemistry. The resulting eigenvalue data shows the motion stiffness and needed energy for the docked complex. Immuno-reactivity analysis through serological estimation is the initial step in evaluating vaccine candidates [85].

The recombinant protein’s expression needs to be in an appropriate host. The expression systems of *E. coli* have been determined for the development of recombinant protein [86]. The optimization of the codon was carried out with a perspective to gain the maximum expression level of our recombinant protein-based vaccine in the K12 strain of *E. coli*. Both the GC-content (45%) and codon adaptation index (CAI) showed the maximum level of potential for protein expression level in the pathogen. Improving the protein’s stability is a major goal in different mechanical as well as biomedical applications. In the present investigation, we have performed disulphide engineering of the MEV structure to enhance the thermostability of the protein. The immune-simulation results showed consistency with conventional immune responses. Following repeated exposure to the antigen, immune responses were improved overall. Furthermore, the result represents a high concentration of TH cells, and thus an effective synthesis of Ig that assists in humoral immune responses. The activity of macrophages and dendritic cells was adequate in this study.

However, the MEV constructed in this present study possesses outstanding properties, as it utilized entire proteome of *C. perfrigens* (ATCC 13124/DSM 756/JCM 1290/NCIMB 6125/NCTC 8237/Type A) to hierarchize identified epitopes that are conserved from its most virulent and important proteins, which is advantageous over reported and traditional vaccines, e.g., it (i) contains epitopes of HTL, LBL, and CTL from highly antigenic proteins, hence may have the ability to initiate humoral and cellular immunity in the host body; (ii) it includes multiple epitopes that focus on various HLAs, and recognize different receptors of T-cells that might be effective in a large population; (iii) a single MEV may contain several targeted proteins as it is concerned with diverse protein immunogenic areas which tend to be combined into one fragment of peptide, and so enhance its effectiveness; (iv) autoimmune diseases can be reduced because human proteins cover epitopes and the remaining undesirable proteins are discarded; (v) lasting immunity might be provided to the host through such kinds of vaccines; (vi) when these vaccines are used orally, sublingually, or intranasally, they might boost immunological responses in mucosa, preventing pathogen entry into the host body through production of host-defensive T- and B- cells in the mucosal and systematic surroundings. Therefore, in the future, these vaccines can become a key tool in fighting bacterial infections. In this research, since the designed multiple epitope vaccine includes B-cells, CTL, and HTL, along with an appropriate adjuvant, it can boost innate immune responses in the host body, thus making it an excellent and appropriate candidate for the development of a vaccine against *C. perfrigens*.

## 5. Conclusions

*C. perfringens* is an emergent bacterium that is involved in severe food poisoning, enterocolitis, histotoxic infections, and gangrene in humans and animals, and has therefore become a keen interest. This investigation gave insight into *C. perfringens*’ important targets for the development of a vaccine, by utilizing the benefits of reverse vaccinology, immuno-informatics, and subtractive genomics techniques. Epitopes of both B- and T-cells retrieved from proteins of *C. perfringens* were selected for MEV development to develop a true immune response. We consider that our MEV will possibly generate humoral and cell-mediated immune reactions. The interaction and binding potentials among receptors and vaccine proteins were stable and maximal. Moreover, immune simulations also showed effective immune responses in real life. The current study is an integrated computational-based pipeline, thus the single limitation in this investigation is the need for further lab work to validate its safety and efficacy.

## Figures and Tables

**Table 1:** CTL selected for MEV vaccine.

CTL	Antigenicity	Allergenicity	Toxicity	Acc No.	Alleles
**KNKKNDPNR**	0.9965	NP	Non-toxic	Q0TMN1	HLA-A*31:01 HLA-E*01:03 HLA-A*30:01 HLA-C*12:03 HLA-B*58:02
**CTECKQRNY**	1.1365	NP	Non-toxic	Q0TMN1	HLA-A*01:01 HLA-A*30:02 HLA-A*29:02 HLA-A*25:01 HLA-A*26:01
**DPNRIEMKK**	1.0997	NP	Non-toxic	Q0TMN1	HLA-B*35:03 HLA-A*68:01 HLA-B*53:01 HLA-B*18:01 HLA-A*11:01
**ENDDVVMDN**	0.7866	NP	Non-toxic	A0A0H2YRY0	HLA-C*05:01 HLA-C*08:02 HLA-C*07:01 HLA-C*04:01 HLA-C*12:03
**KDAIRIKAC**	1.2592	NP	Non-toxic	A0A0H2YRY0	HLA-B*14:02 HLA-B*40:02 HLA-B*44:02 HLA-B*58: HLA-C*14:02
**VVMDNGIKI**	1.1108	NP	Non-toxic	A0A0H2YRY0	HLA-B*51:01 HLA-A*02:06 HLA-C*06:02 HLA-A*02:01 HLA-A*23:01 HLA-A*32:01
**ILGKKFKIY**	0.9003	NP	Non-toxic	A0A0H2YRY0	HLA-A*30:02 HLA-B*08:01 HLA-A*29:02 HLA-E*01:03 HLA-E*01:01
**NSDKDAIRI**	1.1108	NP	Non-toxic	A0A0H2YRY0	HLA-C*08:02 HLA-C*15:02 HLA-A*01:01 HLA-C*12:03 HLA-B*38:01 HLA-B*39:01
**SGAGLNIEV**	2.9786	NP	Non-toxic	A0A0H2YRY0	HLA-A*68:02 HLA-A*02:06 HLA-A*02:01 HLA-B*39:01 HLA-B*46:01
**GAGLNIEVV**	2.6123	NP	Non-toxic	A0A0H2YRY0	HLA-C*12:03 HLA-B*46:01 HLA-C*15:02 HLA-C*07:01 HLA-B*14:02
**DVVMDNGIK**	0.7657	NP	Non-toxic	A0A0H2YRY0	HLA-A*68:01 HLA-A*25:01 HLA-A*11:01 HLA-A*03:01 HLA-B*18:01
**KACSSGAGL**	2.0380	NP	Non-toxic	A0A0H2YRY0	HLA-B*48:01 HLA-A*30:01 HLA-B*58:01 HLA-C*15:02 HLA-B*57:01
**NTILGKKFK**	0.7983	NP	Non-toxic	A0A0H2YRY0	HLA-A*68:01 HLA-A*11:01 HLA-A*30:01 HLA-A*03:01 HLA-A*31:01 HLA-A*25:01
**VTDKKISHF**	0.686	NP	Non-toxic	A0A0H2YRY0	HLA-B*58:02 HLA-A*01:01 HLA-C*05:01 HLA-A*23:01 HLA-B*58:01 HLA-C*04:01
**MKARELKEL**	0.5162	NP	Non-toxic	Q0TMQ4	HLA-A*23:01 HLA-B*58:01 HLA-C*04:01 HLA-A*32:01 HLA-B*15:01 HLA-B*46:01
**REEELKIEQ**	1.5844	NP	Non-toxic	Q0TMQ4	HLA-B*40:01 HLA-B*18:01 HLA-B*44:03 HLA-B*48:01 HLA-B*44:02 HLA-C*05:01
**KAELFNLRF**	2.1029	NP	Non-toxic	Q0TMQ4	HLA-C*05:01 HLA-A*32:01 HLA-B*58:01 HLA-B*57:01 HLA-A*01:01 HLA-A*24:02

**Table 2:** HTL epitopes for MEV vaccine.

HTL	ALLELES	Antigenicity	P-ID	Toxicity
LEYKNTILGKKFKIY	HLA-DRB5*01:05, HLA-DPA1*02:01/DPB1*05:01, HLA-DRB1*11:01 HLA-DRB1*13:07 HLA-DPA1*03:01/DPB1*04:02	0.9003	A0A0H2YRY0	Non-toxic
DATLEYKNTILGKKF	HLA-DRB5*01:05 HLA-DRB1*08:17 HLA-DRB1*08:06 HLA-DRB5*01:01 HLA-DRB5*01:01	0.6102	A0A0H2YRY0	Non-toxic
DNSDKDAIRIKACSS	HLA-DQA1*01:02/DQB1*06:02 HLA-DRB1*11:01 HLA-DRB1*12:01 HLA-DQA1*05:01/DQB1*03:01 HLA-DQA1*03:01/DQB1*03:02	1.2464	A0A0H2YRY0	Non-toxic

**Table 3:** B-cell epitopes for MEV.

B-Cell Epitopes	Protein Acc. No	Antigenicity	Allergenicity	Toxicity	Score
ECKQRNYNTMKNKKND	Q0TMN1	1.2041	Non-allergen	Not toxic	0.94
VTLACTECKQRNYNTM	Q0TMN1	1.0367	Non-allergen	Not toxic	0.81
EQRENDDVVMDNGIKI	A0A0H2YRY0	1.0655	Non-allergen	Not toxic	0.89
MLDNSDKDAIRIKACS	A0A0H2YRY0	0.8408	Non-allergen	Not toxic	0.75
DATLEYKNTILGKKFKI	A0A0H2YRY0	0.6620	Non-allergen	Not toxic	0.66

**Table 4:** Details of docking complex TLR-4 with MEV.

Parameters	MEV-TLR4
HADDOCK score	84.2 ± 23.3
Cluster size	15
RMSD from the overall lowest-energy structure	0.7 ± 0.4
Van der Waals energy	−150.8 ± 8.6
Electrostatic energy	−538.7 ± 93.2
Desolation energy	−5.3 ± 2.2
Restraints violation energy	3480.5 ± 117.1
Buried surface area	5373.2 ± 389.3
Z-score	−2.1

## Data Availability

The data presented in this study are available within the article.
